# Activation of Marck-like Genes and Proteins During Initial Phases of Regeneration in the Amputated Tail and Limb of the Lizard *Podarcis muralis*

**DOI:** 10.3390/jdb13020012

**Published:** 2025-04-14

**Authors:** Lorenzo Alibardi

**Affiliations:** 1 PAM, Comparative Histolab Padova, 35100 Padova, Italy; lorenzo.alibardi@unibo.it; 2 Department of Biology, University of Bologna, Via Selmi 3, 40126 Bologna, Italy

**Keywords:** lizard, tail, limb, wound healing, dedifferentiation molecules

## Abstract

Molecules involved in the activation of regeneration in reptiles are almost unknown. MARCK-like proteins are indicated to activate regeneration in some amphibians and fish, and it would be important to know whether this is a general process also present in other vertebrates. To address this problem, the present study reports the immunolocalization of a MARCK-like protein in injured tissues of a lizard. Bioinformatics and immunofluorescence after 5BrdU administration, and detection of MARCK-like proteins, have been performed on regenerating tail and limb of the lizard *Podarcis muralis*. Transcriptome data indicate up-regulation of MARCKS and MARCK-like1 expression in the initial regenerating tail and limb blastemas, supporting their involvement in the activation of regeneration in both appendages. Immunofluorescence for 5BrdU shows numerous proliferating cells in the blastemas of both appendages. Immunolocalization of a MARCK-like protein, using an antibody generated against a homologous protein from the axolotl, shows that the wound epidermis, nerves, and myotubes accumulate most of the protein in the limb and tail. MARCK-like immunolabeling is also detected in the regenerating spinal cord of the tail. The study indicates that, although the limb later turns into a scar, the MARCK-like protein is also up-regulated in this appendage, like in the regenerating tail. These results indicate that the initial reaction to an injury in lizards, an amniote representative, includes some triggering processes observed in amphibians and fish (anamniotes), with the activation of MARCK-like genes and proteins. This suggests that a MARCK-like-dependant mechanism for tissue repair is likely activated during the initial phases of vertebrate wound healing.

## 1. Introduction

Among amniotes, a scarce reparative capability is elicited after extensive injury or loss of body appendages such as limbs or tails [1,2]. In cases of survival, the damaged or lost appendage is not regenerated but only repaired by an extensive and non-functional scar. An exception to this negative outcome is represented by the regenerating tail of lizards, which can vary from a 20–90% return to its original size [3,4,5,6,7,8,9,10]. Extensive studies on the expression of numerous genes and proteins that are activated during the process of tail regeneration in lizards have discovered some of the genes and proteins involved in the process [11,12,13,14,15,16,17]. Among these genes are those for c-myc, telomerase proteins, and the extensive activation of the wnt-pathways through a number of its ligands (wnt1, wnt2b, wnt5a, wnt5b, wnt6) [11,12]. These studies indicate that, as for other body appendages in vertebrates, classical signaling proteins and growth factors are also activated during lizard regeneration, as part of a general wound healing process.

Aside from c-myc and telomerase activation, little is known about other factors that trigger and later sustain the regeneration of the tail of lizards, which represent useful models for analyzing organ regeneration in amniotes. Important information has been derived from the analysis of the transcriptome in the wall lizard, *Podarcis muralis* [12]. Particularly important is the up-regulation of genes known in other models of vertebrate regeneration, fish, and amphibians, to stimulate the initiation of the process after injury of a limb or tail. It is known that a process of cell dedifferentiation takes place during the first days post-trauma in a tail, fin, or limb in fish and amphibians, and also in the amputated tail stump of lizards [10,18,19,20,21]. A process of cell reprogramming appears necessary, together the recruitment of stem cells from various tissues of lizards, in order to create a critical mass of mesenchymal-like cells that form a regenerative blastema [14,15].

Among these factors activated during initial stages of appendage regeneration are proteins such as MARCK-like (Myrystoylated Alanine-Rich C Kinases Substrate-like protein), whose genes are also up-regulated during tail and limb regeneration in the wall lizard *P. muralis* [12,18]. It is known that, in amphibians, MARCK-like proteins are activated during the early phases of appendage regeneration, likely acting as initiating signals for tissue injury and successive regeneration [22]. In particular, the MARCK1 gene and its protein are indicated as initial stimulators for limb regeneration in the urodele axolotl [22]. No information is available on the prevalent tissue localization of MARCK-like proteins in the regenerating tissues of lizards. The present study was carried out to detect whether similar signaling molecules are involved in the activation of regeneration in lizard appendages, as an amniote representative. This present bioinformatics and immunohistochemical study has been centered on the expression and localization of MARCK-like proteins in cells of early regenerating tails and scarring limbs of the lizard *P. muralis*.

## 2. Materials and Methods

### 2.1. Sampling and Tissue Preparation

A total of 21 adult specimens of the common wall lizard (*Podarcis muralis*) were utilized in the present survey, using new tissue regions that were fixed and embedded in previous studies (details in [16,18,23]. The experiments followed Italian regulations on animal care and handling (art. 5, DL 116/92). As reported in detail in the referred papers, the tail was autotomized at about one-third distal, while the hindlimb was amputated under hypothermic conditions at a level corresponding to about half of the femur.

After tail autotomy, regenerating tails of 1–3 mm in length and hindlimbs of 0.5–1.0 mm were collected and fixed (13–18 days post-amputation). Sampled tissues from the regenerating tail from previous studies were selected and further utilized in the present study. They included three pre-blastema tissues at 8–10 days post-autotomy (about 0.5 mm), three blastemas of 1.0–1.5 mm, three coniform blastemas of 2 mm, and two elongating cones of 3.0–3.5 mm in length. Sample tissues from regenerating limbs were also selected, including two pre-blastemas at 9–10 days post-amputation, two blastemas at 13–14 days (not completely re-epithelialized), and two at 17–18 days post-amputation (re-epithelialized, with partial scabs still present). To detect cell proliferation in regenerating tail and limb tissues, another four lizards with regenerating tails and limbs (approximately two weeks post-injury) were injected intra-peritoneum with a Ringer solution of 5-Bromo-deoxy-Urydine (5BrdU, Sigma, San Louis, MI, USA) at 50 μg/g body weight, 3–4 h before sacrifice.

The small stumps of the limbs with the blastema (about 1 mm total) were fixed whole, while the regenerating tails of 1–3 mm were halved and fixed for allowing better and rapid fixation. The tissues were fixed for 6–8 h in 4–5% paraformaldehyde in 0.1M phosphate neutral buffer at approximately 4 °C Tissues were then rinsed in the same buffer for half of an hour, progressively dehydrated with ethanol, clarified in xylene, and finally embedded in wax. Wax-embedded tissues were sectioned at 6–9 μm thickness using a microtome. The derived sections were collected on gelatin-chromoalume-coated slides and dried over a hot plate at 40–50 °C for 2–3 h.

### 2.2. Microscopical Methods

Some sections were stained with 0.5% toluidine blue or hematoxylin-eosin for general histology. For immunolabeling, other sections were de-waxed, rinsed in distilled water, and then pre-incubated at room temperature for 30 min in 0.05 M Tris buffer (pH 7.6) containing 2% BSA and 5% NGS to block non-specific antigenic sites present in the tissues. After rinsing in the buffer, the sections were incubated overnight at 4 °C with primary antibodies (against 5BrdU or against a MARCK-like protein). The mouse monoclonal anti-5BrdU (G3G4, DSHB) was purchased from the Developmental Studies Hybridoma Bank (DSHB, University of Iowa, Iowa City, IA, USA) and utilized at 1:100 dilution (*v*/*v*) in the buffer. For the MARCK-like immunostaining, a rabbit antibody produced against the axolotl protein was generously donated by Dr. Takuji Sugiura (DFG Research Center for Regenerative Therapies, Dresden, Germany; see [22]), and was utilized at 1:500 dilution in buffer. In some control sections, primary antibodies (from mouse or rabbit) were omitted from the incubating solution (negative controls).

After rinsing in Tris buffer for about 15 min in two changes, the sections were incubated for 1 h at room temperature with Tetramethyl Rhodamine Isothiocyanate (TRITC)-conjugated anti-mouse or anti-rabbit IgGs (Sigma, San Louis, MI, USA, diluted 1:200), and rinsed in buffer. Some sections were also counterstained for 10 min with the blue fluorophore DAPI (diluted 1:1000 *v*/*v* in buffer) for nuclear staining. This nuclear staining also improved the detection of non-specific vs. specific fluorescence. After rinsing in buffer for 20 min with two changes, the stained sections were mounted in anti-fading medium (Fluoromount, Sigma, San Louis, MI, USA) and observed under a fluorescence microscope using specific filters for TRITC and DAPI. Pictures were taken with a digital camera and digitalized into a computer using Adobe Photoshop, version 8.0.

## 3. Results

### 3.1. Histology and 5BrdU-Immunolabeling in the Tail and Limb Initial Blastemas

Details of the progressive phases of tail and limb regeneration in *P. muralis* (and *P. sicula,* now *P. siculus*) have been extensively reported [5,14,15,18,23], and only the essential descriptions needed for following the immunolabeling observations are reported here.

**Figure 1:**
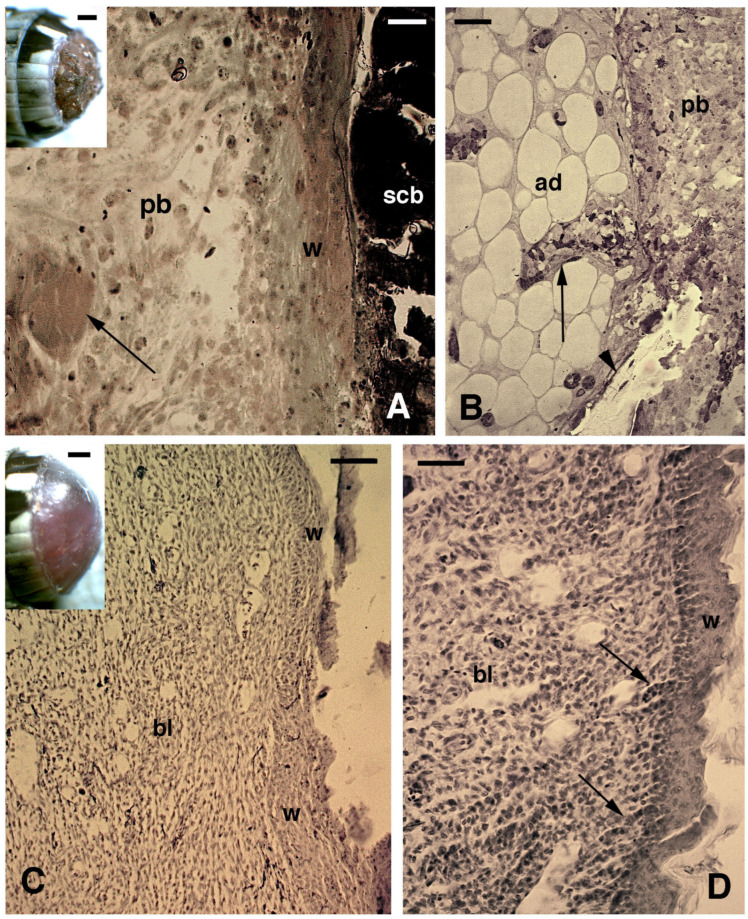
Histology of early stages of tail regeneration ((**A**,**B**), toluidine blue staining; (**C**,**D**), hematoxylin-eosin stain). (**A**) Regenerating sample at about 10 days post-autotomy (the inset, bar = 0.5 mm, shows a stump at 4 days post-autotomy). Underneath the dark scab, a “tongue” of elongated wound keratinocytes is present, covering the loose pre-blastema tissue and a muscle fragment (arrow). Bar = 20 μm. (**B**) Detail of the stump at about 10 days, showing the adipose tissue covered by blastema cells, some blood cells within a vessel (arrow), and a piece of vertebral bone (arrowhead). Bar= 20 μm. (**C**) Blastema of about 1 mm (similar to the inset, bar = 0.5 mm), showing the thick wound epidermis and the loose mesenchymal tissue. Bar = 40 μm. (**D**) Close-up view of the apical blastema of about 1.5 mm, focusing on the irregular wound epidermis where some small papillae suggesting EMT (Epithelial Mesenchymal Transition, arrows) are observed. Bar = 20 μm. Legends: ad, adipose cells/tissue; bl, blastema; pb, pre-blastema; scb, scab; w, wound (regenerating) epidermis.

**Figure 2:**
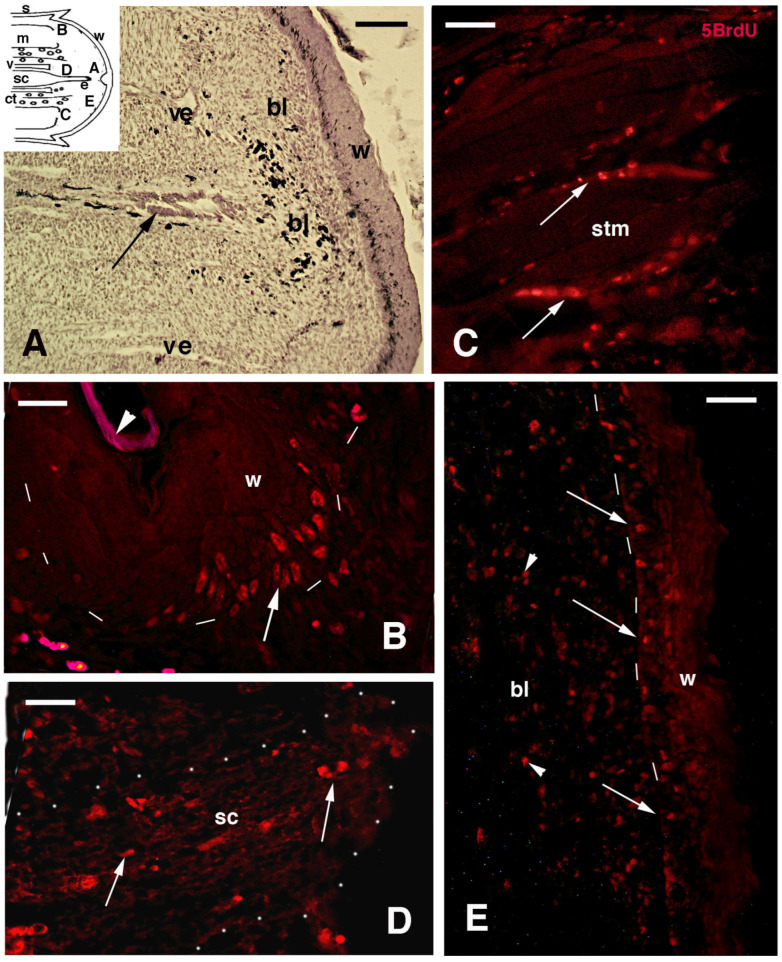
Histological image ((**A**), hematoxylin-eosin stain) and 5BrdU-immunofluorescent images (TRITC, (**B**–**E**)) of early blastemas (about 14 days of regeneration). (**A**) Central part of a blastema of about 2 mm, focused on the central area with the axial ependyma (arrow). The inset shows the drawing of a blastema with indicative locations of the following images. Bar = 50 μm. (**B**) Proximal wound epidermis in continuation with that of the stump scale, where numerous labeled cells are present (arrow). The arrowhead indicates the nonspecifically fluorescent corneous layer of the proximal stump scale. Bar = 10 μm. (**C**) Detail of stump muscles with labeled cells in the inter-muscle connective (arrows), or associated with the fiber (likely satellite cells). Bar = 20 μm. (**D**) Detail of the transected spinal cord (outlined by dots), containing sparse labeled cells (arrows). Bar = 20 μm. (**E**) Apical wound epidermis with labeled cells (arrows). Sparse labeled cells (arrowheads) are present in the blastema mesenchyme. Bar = 20 μm. Legends: bl, blastema; ct, connective and fat tissue; m, muscles (stump); s, scale; sc, spinal cord stump; stm, stump muscles; v, vertebra; ve, blood vessel; w, wound epidermis. Dashes underline the epidermis.

After tail loss through autotomy (a natural auto-amputation mechanism), the exposed tissues of the stump formed a clotting surface that was re-epithelialized within 7–12 days by the migration of elongated keratinocytes crawling underneath the scab (Figure 1A). During this period—the pre-blastema phase—numerous isolated cells of mesenchymal-shape (irregular), mixed with red and white blood cells, were accumulated over stump tissues. The latter included mainly muscles, connectives, and fat tissues, and some free cells were also accumulated over the transected vertebrae and spinal cord (Figure 1B). Mesenchymal and fibroblast cells largely prevailed over blood cells in the tail pre-blastema at 7–12 days post-amputation. When a regenerative blastema was formed at 14–16 days post-amputation, it was mainly composed of loose mesenchymal-like connective tissue with various blood vessels and sparse pigment cells. These tissues were covered with a multilayered wound epidermis (Figure 1C,D). Very early in the process, the mesenchymal blastema was colonized by the regenerating spinal cord that formed an apical ampulla, located in the middle of the blastema. The spinal cord consisted of ependymal cells surrounded by a few axons (Figure 2A). In lateral areas of the conical blastema, 2–3 mm in length and close to the stump tissues, some aggregation of fusiform and small bipolar-shaped cells were seen. The latter formed the so called pro-muscle aggregates and anticipated the formation of new muscles (see later immunolabeling). Immunolabeling for 5BrdU showed numerous immunolabeled cells (nuclei) localized in the proximal wound epidermis, in continuity with stump scales. Labeled cells were also detected in the intermuscular connectives and identified as satellite cells in muscle bundles of the stump (Figure 2B,C). Sparse 5BrdU-labeled cells were also located in the transected spinal cord, surrounding connective laminae, within the blastema, and in the wound epidermis (Figure 2D,E).

**Figure 3:**
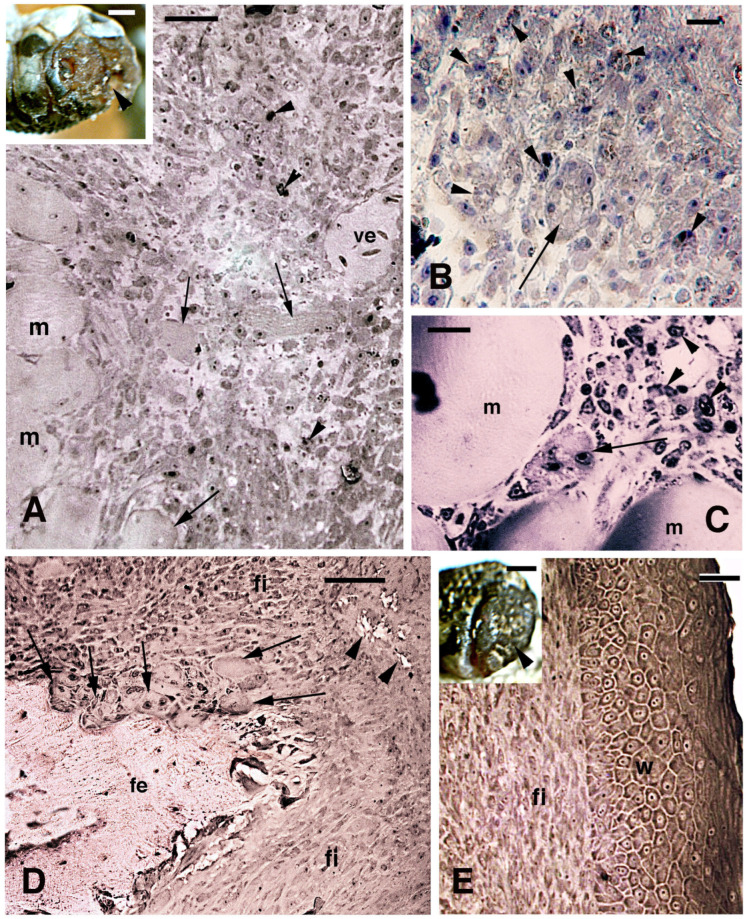
Limb blastema and its histology at 10–18 days (toludine blue stain). (**A**) Central region of a pre-blastema at 10 days post-amputation (the inset, bar = 0.5 mm, shows the surface of the stump photographed at 7 days post-amputation, arrowhead, still covered by a clot). Numerous free mesenchymal-like cells (irregular) and fibroblasts (fusiform) are present in continuation with stump muscles and their fragments (arrows). Arrowheads indicate sparse dark phagocytes. Bar = 20 μm. (**B**) Detail of apical pre-blastema close to the surface not yet covered by a wound epidermis at 10 days post-amputation. Numerous phagocytes (arrowheads) are present among free mesenchymal-like cells, and also some multinucleated bodies (arrow), representing either muscle fragments or a giant cell. Bar = 10 μm. (**C**) Detail of a multinucleated body (arrow), likely a muscle fragment, at 13 days post-amputation, surrounded by mononucleated cells (immune, arrowheads). Bar = 10 μm. (**D**) Apical region of amputated femur with resorbing surface at 14 days post-amputation, where numerous poly-nucleated osteoclasts are present (arrows). A dense connective tissue surrounds the bone fragments distally (arrowheads). Bar = 20 μm. (**E**) Fibrous outgrowth covered from a thick wound epidermis at 18 days post-amputation (the inset, bar = 0.5 mm, shows the surface still largely covered by a scab, arrowhead). Bar = 10 μm. Legends: fe, femur; fi, fibrous connective tissue (scar); m, muscles; ve, blood vessel; w, wound epidermis.

**Figure 4:**
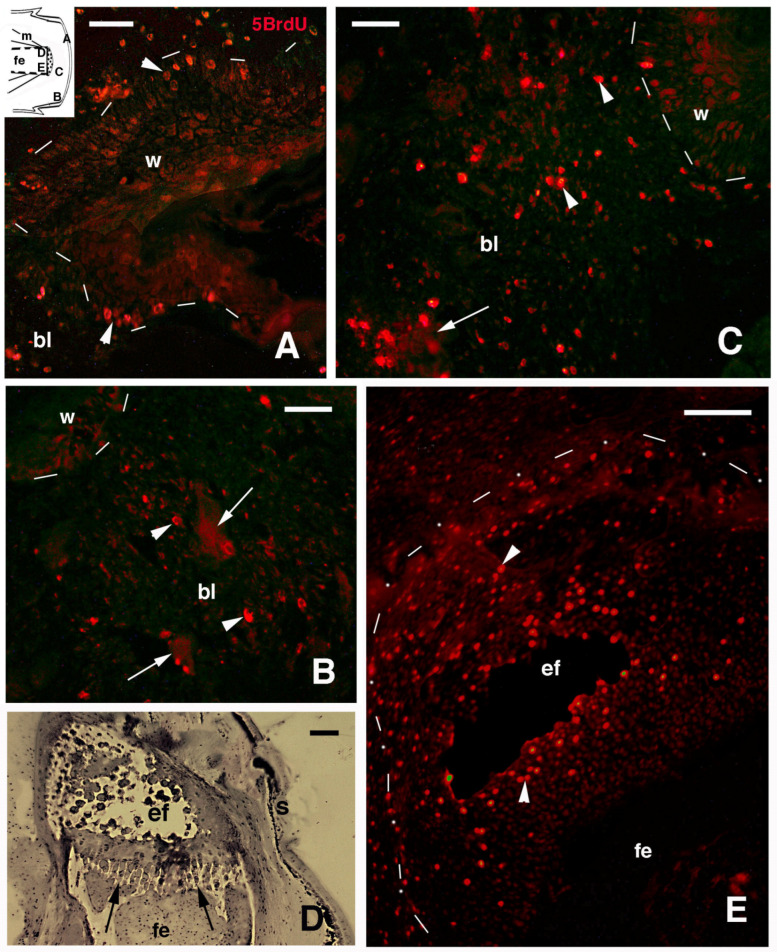
Immunofluorescence for 5BrdU (**A**–**C**,**E**) and histology (**D**) of amputated limb. (**A**) Apical blastema with epidermal fold 14 days post-amputation (arrowheads indicate labeled keratinocytes). Bar = 10 μm. The inset shows a drawing of a pre-blastema with indicative locations of the following images. (**B**) Pre-blastema at 14 days with sparse labeled cells (arrowheads) and labeled tissue fragments (arrows), likely of muscle derivation. Bar = 10 μm. (**C**) Central region of pre-blastema at 14 days post-amputation showing numerous labeled cells (arrowheads) and a labeled tissue fragment (arrow). Bar = 10 μm. (**D**) Detail of a femur epiphyses with growth plate (arrows) at 10 days post-amputation. The distal direction to the stump surface is below. Toluidine blue stain. Bar = 20 μm. (**E**) Epiphyses (outlined by dots and dashes) containing numerous labeled cells (arrowheads) at 14 days post-amputation. The distal direction to the stump surface is below to the right. Bar = 20 μm. Legends: bl, (pre-)blastema; ef, femur epiphisis; fe, femur; m, muscles; w, wound epidermis. Dashes underline the wound epidermis.

In the injured limb at 13–14 days post-amputation, numerous small cells populated the region over the injured stump tissues. The latter were not completely covered by a multilayered wound epithelium (Figure 3A). Fragments of muscles and numerous blood cells (red and white, including granulocytes and mononucleated macrophages and lymphocytes) were present in the pre-blastema also at 17–18 days post-amputation. This indicated that a strongly inflamed connective tissue was present on the limb stump (Figure 3B,C). Large muscles fragments, still multinucleated, were frequently encountered among the injured muscles, in continuation with the pre-blastema mass of isolated cells (Figure 3C). The transected femur at 13–14 days post-amputation was surrounded by osteoclasts and by small cells of fibroblast shape (fusiform) or more irregular, including mononuclear (immune) cells (Figure 3D). At 17–18 days post-amputation, numerous inflammatory cells were still mixed with a population of fusiform fibroblasts. The latter cells were densely aggregated underneath the thick wound epidermis where a stratum corneum was present (Figure 3E). Already at this stage, the pre-blastema appeared prevalently fibrotic, anticipating its development into scar tissue in the following days (details in [5,16,23]). Despite the latter fate, also in the pre-blastema of the limb at 13–18 days post-amputation, numerous 5BrdU-labeld cells were observed (Figure 4). The apical wound epidermis in some samples did not completely cover the stump. The regenerating keratinocytes contained sparse immunolabeled nuclei with different immunofluorescence intensities (Figure 4A). Sparse labeled cells were also detected in the pre-blastema, located underneath the wound epidermis, or among the injured muscles and inter-muscle connective tissue (Figure 4B,C). The transected femur and also its epiphyseal portion that articulates with the tibia appeared surrounded and invaded by numerous 5BrdU-labeled cells. This was particularly evident at 17–18 days post-amputation (Figure 4D,E). Therefore, both injured and proximal uninjured bones featured 5BrdU-labeled cells after limb amputation during the initial 2–3 weeks post-trauma (see [16]).

### 3.2. Bioinformatics Analysis

**Figure 5:**
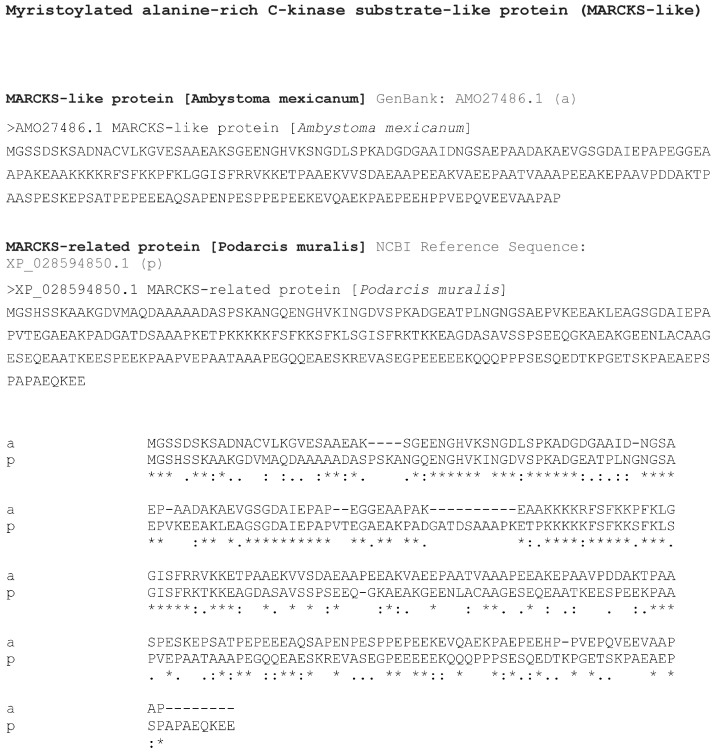
Clustal-W comparison between the axolotl MARCKL-like proteins (a) and a MARCK-like 1-related protein of *P. muralis* (l), detected using protein BLAST. A higher identity is detected in amino acid regions present in the initial 120 amino acid N-terminal. Accession numbers are indicated for each protein. Stars represent identities (same amino acid), colons indicate substitutive but conservative replacements (amino acids with similar chemical-physical characteristics such as 3D-shape, size, and solubility), and dots indicate semi-conserved substitutions (amino acids with similar size but different polarity).

From the transcriptome data [12,18], an up-regulation of MARCKS (myristoylated alanine-rich protein kinase C substrate) transcripts was noted in both the regenerating tail and limb blastemas of *P. muralis*. In the early regenerating tail blastema, a tail-exclusive MARCKS gene appeared up-regulated 4.1 folds in comparison to the normal tail. Another gene, a MARCK-like1 gene, in the regenerated tail blastema was expressed 4.2-fold compared to normal tail tissues. Finally, the same MARCK-like 1 gene was also overexpressed in the limb, 4.4-fold compared to normal limb tissues. BLAST analysis https://blast.ncbi.nlm.nih.gov/Blast.cgi?PAGE=Proteins), using the axolotl sequence, detected MARCK-like proteins in the genome of *P. muralis* present in the NIH-database (Figure 5). Comparison of the lizard with the axolotl sequence, using the CLUSTAL-W program (https://www.genome.jp/tools-bin/clustalw), showed extensive identity between the two proteins (Figure 5), supporting a likely cross-reactivity for immunolabeling.

### 3.3. MARCK-like Immunolocalization

**Figure 6:**
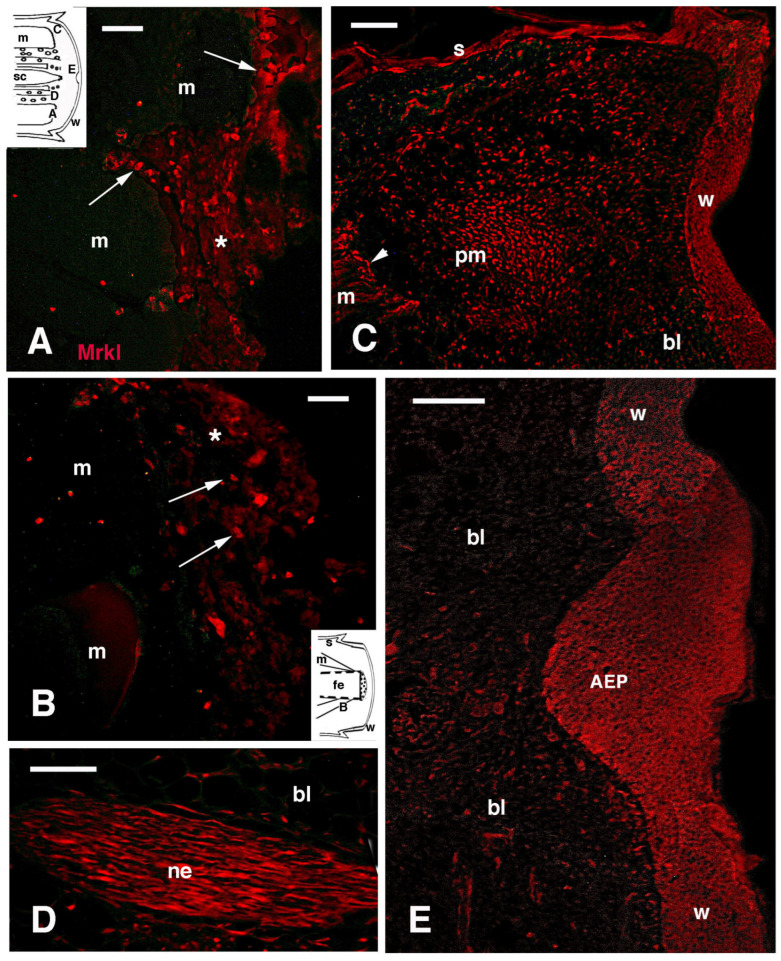
TRITC immunofluorescence for MARCK-like protein in the tail blastema (**A**,**C**–**E**) and limb (**B**) pre-blastema. (**A**) Detail on the surface of stump muscles, evidencing immunofluorescent cells (arrows) and weaker labeling in the surrounding extracellular matrix (asterisk). The inset shows a drawing of a blastema with indicative locations of the following images. Bar = 10 μm. (**B**) Detail on the surface of stump muscles in a limb at 11 days post-amputation. Arrows point to labeled cells while the asterisk indicates weak labeling in the extracellular matrix. Bar = 10 μm. The inset shows a drawing of a pre-blastema with the indicative location of the image. (**C**) Lateral area of early tail blastema at 13 days post-amputation, with immunolabeled wound epidermis and pro-muscle aggregate close to distal terminals of stump muscles (arrowhead). Bar = 40 μm. (**D**) Intensely immunofluorescent regenerating nerve located inside a 2 mm long blastema. Bar = 20 μm. (**E**) Central apical blastema with immunolabeled Apical Epidermal Peg. Sparse blastema cells are also weakly labeled. Bar = 40 μm. Legends: AEP, Apical Epidermal Peg; bl, blastema; fe, femur; m, muscles (stump); ne, regenerating nerve; pm, pro-muscle aggregate (myoblasts condensation); sc, spinal cord (stump); s, scale (epidermis); w, wound epidermis.

Due to the small availability of the MARCK-like antibody, most observations were done on tail sections. The immunofluorescence, using only TRITC labeling for MARCK-like protein, was located only on the surface of the stump muscles in the tail and limb at 13–18 days post-amputation. Uneven immunofluorescence was observed in sparse cells, in some nuclei, and—less intensely—also in the extracellular matrix among inter-muscle connective tissues (Figure 6A,B). In the regenerating early blastemas of 1–3 mm in length, immunofluorescence was mainly detected over the thick wound epidermis and in the repairing muscles or even in the pro-muscle aggregates (Figure 6C). Wound keratinocytes were labeled only in their cytoplasm and along the cell perimeter, while almost no nuclear immunolabeling was seen. Additionally, sparse nerves entering the tail blastema appeared strongly immunolabeled (Figure 6D). Sparse blastema cells—mesenchymal or fibroblasts of unknown tissue derivation—were not labeled or some were weakly labeled in comparison to the wound epidermis (Figure 6C,E).

**Figure 7:**
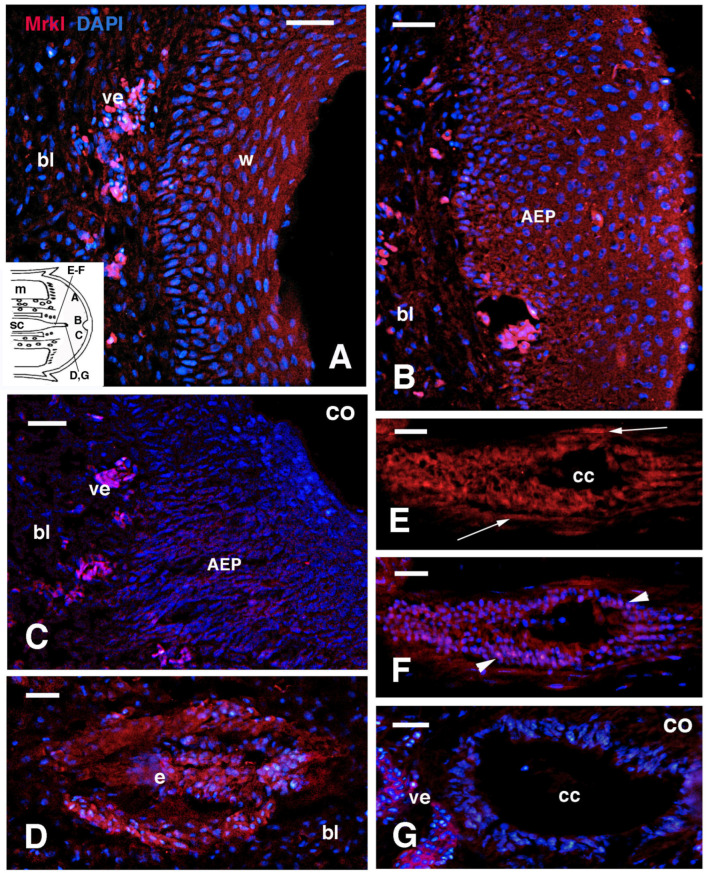
Double immunolabeling for MARCK-like in blastemas of 1.5–2.0 mm in length. (**A**) Immunopositive apical wound epidermis. Bar = 10 μm. The inset shows a drawing of a blastema with indicative locations of the following images. (**B**) Immunolabeled Apical Epidermal Peg. Bar = 10 μm. (**C**) Immunonegative control section. Bar = 10 μm. (**D**) Immunofluorescent apical ependymal ampulla (mainly tangentially sectioned). Bar = 10 μm. (**E**) Ependymal tube immunofluorescent for MARCK-like protein. Arrows indicate nerves. Bar = 10 μm. (**F**) Same image counterstained with DAPI. Arrowheads indicate double-labeled nuclei. Bar = 10 μm. (**G**) Immunonegative apical ependymal ampulla control. Bar = 10 μm. Legends: AEP, Apical Epidermal Peg; bl, blastema; cc, central canal (ependymal lumen); e, ependyma; m, muscle; sc, spinal cord; ve, blood vessels (autofluorescent pinkish); w, wound epidermis.

Using DAPI (Sigma, San Louis, MI, USA) for nuclear counterstaining in addition to TRITC, the observations confirmed the immunolocalization in regenerating tail blastemas, 1–3 mm in length. The wound epidermis was well labeled, while blastema cells appeared mostly unlabeled, while a weak immunofluorescence was present in the extracellular matrix (Figure 7A,B). No nuclear labeling was detected in either apical and lateral wound epidermis covering the cone-shaped blastema. Control sections were unlabeled or showed faint, irregular fluorescence (Figure 7C). The apical ependymal ampulla, also present in elongating blastemas, appeared more immunofluorescent in comparison to the surrounding blastema cells (Figure 7D). Most of the ependymal epithelium located in proximal regions close to the spinal cord of the stump tissues also showed immunolabeling (Figure 7E,F). Contrary to the wound epidermis, some ependymal cells were also labeled in their nuclei (arrowheads in Figure 7F). In control sections, feeble to absent immunolabeling was observed in the ependymal epithelium (Figure 7G). Numerous blood vessels in the blastema appeared autofluorescent with a pinkish color, as confirmed in controls (Figure 7C,G).

**Figure 8:**
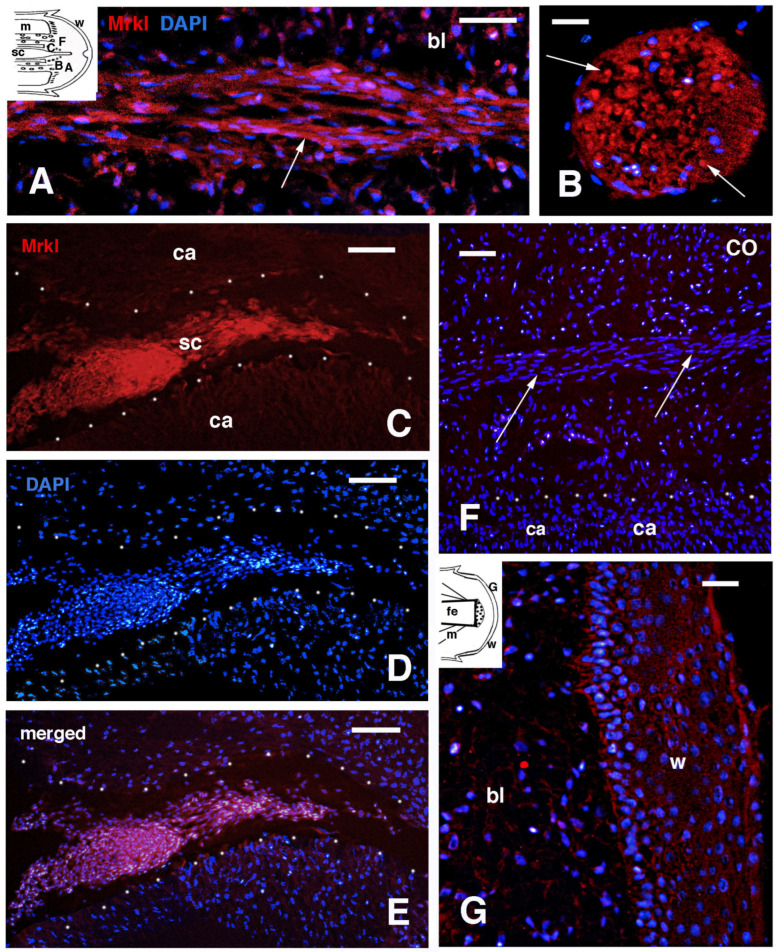
Double immunolabeling for MARCK-like in tail blastemas of about 3 mm in length (**A**–**F**) and in a limb of 17–18 days post-amputation (**G**). (**A**) Immunofluorscent axons (arrow) in a regenerating nerve. Bar = 20 μm. The inset shows a drawing of a blastema with indicative locations of the following images. (**B**) Cross-sectioned, proximal nerve with labeled axons (arrows). Bar = 10 μm. (**C**) Tangential section of the regenerating proximal spinal cord, close to the stump, and surrounded by the immunonegative cartilaginous tube (regenerating, outlined by dots). The intense MARCK-like immunolabeling derives mainly from the regenerating nerves surrounding the ependyma, where the lumen is not visible. Bar = 20 μm. (**D**) Same section with DAPI. Bar = 20 μm. (**E**) Merged image. Bar = 20 μm. (**F**) Immunonegative control section with a regenerating nerve (arrows). Dots outline part of the regenerating cartilage. Bar = 20 μm. (**G**) Apical blastema of a limb showing weak immunofluorescence in the wound epidermis. Bar = 10 μm. Legends: bl, blastema; ca, regenerating cartilage; sc, spinal cord; w, wound epidermis.

In elongating cones of 3 mm, regenerating nerves appeared with a contrastingly stronger immunolabeling with respect to the surrounding connective and blastema cells (Figure 8A,B). The regenerating spinal cord in more proximal regions of the elongating cones, made of ependymal cells and associated regenerating nerves, was immunolabeled. In contrast, the surrounding cells of the cartilaginous tube appeared immunonegative (Figure 8C,D). In control sections, regenerating nerves were unlabeled for the MARCK-like protein (Figure 8F). Finally, the wound epidermis of the limb at 13–18 days post-amputation also showed some LOW BUT positive immunolabeling, particularly in the peripheral cytoplasm of keratinocytes, but not in their nuclei (Figure 8G).

## 4. Discussion

The present observations detected MARCKS and MARCK-like protein in the initial blastema of the tail and limb, confirming previous indications from the transcriptome of *P. muralis* [12]. Checking the list of genes detected in the transcriptomes so far published from some lizard species, the MARCK gene also appears up-regulated during early stages of regeneration ([11,17], see their supplementary figures). MARCKS and MARCK-like genes and proteins are present in normal tissues of various vertebrates, especially in the nervous system [24,25,26], but become more expressed and immunodetected in developing and regenerating tissues, in particular in nervous tissues [22,27,28,29].

This gene and its coded protein are indicated as one of the key signaling molecules activated at the beginning of limb regeneration in the axolotl [22], and likely also in the South American lungfish [30]. The protein identity and most tissue localizations in lizard tissues recall similar observations previously reported in the axolotl, although no regenerating muscles were detected or reported for this neotenic urodele. In particular, the MARCK-like protein determines the cell cycle re-entry in myotubes of the axolotl [22], and is also involved in myoblast fusion into myotubes in the developing chick [31,32]. Whether the immunolabeling observed here in myoblasts and early myotubes of the lizard may also indicate such a process remains undetermined. The immunolocalization in injured muscles of lizard instead supports previous tract-tracing and ultrastructural observations indicating dedifferentiation, with the liberation of cartilage precursors from the injured vertebrate and myoblasts from lesioned muscle fibers of the tail stump [10,18].

The extracellular labeling noted in some areas of the damaged muscles is interesting, since the protein, in the axolotl, has been hypothesized to be released extracellularly, perhaps also from the wound epidermis [22]. The hypothesized action of MARCK-like release is the stimulation of myotube cycle re-entry [22]. Besides, the sparse MARCK-like cells present at the surface of the stump tissues and in the blastema—whose specific type remains undetermined—may also represent immune cells [25,26]. The latter are known to be present at the early stages in both the tail and especially injured limbs [5,18,23,33,34]. MARCKS has also been implicated in the process of extrusion of secretory vesicles in different cell types, including mucus production from epithelial cells of the bronchus epithelium [35].

In different cells, the MARCKS protein can move from the plasmalemma, to which it is bound in the unphosphorylated form, to the cytoplasm after its phosphorylation by C kinase [36,37]. The protein can even be imported into the nucleus to activate genes stimulating cell proliferation and cytokinesis [36,37]. The nuclear labeling observed here in ependymal cells may also indicate that the MARCK-like protein is involved in the high proliferation known to be present in these cells [3,11,16,38]. The localization of MARCKS in some nuclei of ependymal cells and in migrating neuroblasts has been reported for the developing and regenerating spinal cord in *Xenopus laevis* [29], suggesting also in this case a correlation with intense proliferation. MARCKS has also been localized in ependymal radial cells (tanicytes) in developing mice cortex—glial cells involved in the mobilization and terminal placement of cortical neurons [28].

In the regenerating axons of neurons, the protein is mainly localized in association with GAP43 and the actin cytoskeleton, indicating that it is implicated in the elongation of growth cones [27,29]. In the regenerating blastema and elongating cones of lizards, regenerating nerves are present in association with ependymal cells and are derived from spinal ganglia and motor neurons of the stump spinal cord [7,9,39,40]. Their intense immunolabeling for MARCK-like proteins is very likely due to interaction with actin and its dynamic activity. Finally, the localization in the peripheral cytoplasm of wound keratinocytes, previously seen also in those of the axolotl [22], indicates that the protein is associated with the peripheral actin cytoskeleton. The latter is present in lizard keratinocytes, where it contributes to their movement [41]. A number of studies report that the association of MARCKS or MARCK-like proteins with the actin cytoskeleton in various cell types determines modification of their plasma membrane and promotes cell migration [25,26]. The comparison of MARCK-like immunolabeling between the lizard tail, which regenerates, and the limb, which does not, indicates that the expression of MARCKs is a primary reaction to the injury of tissues. This activation is independent from the final fate of the appendage—successful (tail) or unsuccessful (limb) regeneration.

In conclusion, the detection of MARCK-like immunolabeling in early stages of tail and limb regeneration in a lizard, like in the initial stages of regeneration in anamniotes and mammalian nerve tissues, suggests that this is a general mechanism promoting wound healing in vertebrates.

## Data Availability

The original contributions presented in this study are included in the article. Further inquiries can be directed to the corresponding author.
